# Pembrolizumab-Related Central Nervous System Vasculitis Presenting With Recurrent Ischemic Strokes

**DOI:** 10.7759/cureus.97370

**Published:** 2025-11-20

**Authors:** Fumiaki Henmi, Yoshifumi Ogasawara, Yoshikazu Uesaka

**Affiliations:** 1 Neurology, Toranomon Hospital, Tokyo, JPN; 2 Neurology, The University of Tokyo Hospital, Tokyo, JPN

**Keywords:** black blood imaging, cerebral arteriopathy, cerebral infarction, immune checkpoint inhibitor (ici), immune-related adverse event (irae), non-small-cell lung cancer, pembrolizumab, recurrent stroke, vasculitis, vessel wall imaging (vwi)

## Abstract

Immune checkpoint inhibitors (ICIs) can precipitate immune-related adverse events (irAEs), including rare cerebrovascular complications. We report the case of a 52-year-old man receiving pembrolizumab for non-small-cell lung cancer who developed multiple cerebral infarctions over a short interval. Despite appropriate antiplatelet therapy after the initial event, he experienced three recurrent ischemic strokes, and vascular imaging demonstrated multifocal intracranial arterial stenoses (including the middle cerebral artery). Following discontinuation of pembrolizumab and initiation of corticosteroid therapy, the stenoses regressed, and no further infarctions occurred. The overall clinical-radiologic picture was most consistent with cerebral vasculitis as an ICI-related adverse event. This case highlights that even after long-term ICI therapy, cerebral infarction can occur and may recur within a short period. When this happens, irAEs should be promptly considered. Early ICI cessation together with initiation of corticosteroids should also be considered, because antithrombotic therapy alone may be insufficient.

## Introduction

Immune checkpoint inhibitors (ICIs), such as programmed cell death-1 (PD-1) inhibitors, have significantly improved survival in various malignancies, including advanced non-small-cell lung cancer. However, they can induce a wide spectrum of immune-related adverse events (irAEs), involving multiple organs, including the central and peripheral nervous systems [1,2]. Neurological irAEs (nirAEs) are relatively rare but may present with life-threatening complications, such as cerebral vasculitis and ischemic stroke [3,4]. While isolated cases of ICI-induced cerebral vasculitis have been reported [5], recurrent ischemic strokes associated with progressive intracranial arterial stenosis during long-term ICI therapy remain extremely rare. We report the case of a 52-year-old man with lung adenocarcinoma who developed multiple recurrent ischemic strokes and fluctuating intracranial arterial stenosis during long-term pembrolizumab therapy, which improved after discontinuation of the drug and corticosteroid therapy.

## Case presentation

A 52-year-old man with a history of treated neurosyphilis had no history of hypertension, diabetes, dyslipidemia, or smoking. He underwent left lower lobectomy for stage IVA lung adenocarcinoma (pT1aN2M1a, with pleural dissemination and malignant effusion), followed by adjuvant chemotherapy with carboplatin, pemetrexed, and pembrolizumab. Because of renal dysfunction, the regimen was switched to pembrolizumab monotherapy.

**Figure 1:**
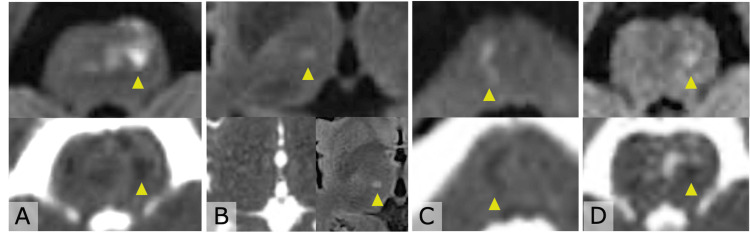
Serial diffusion-weighted MRI imaging (DWI) and apparent diffusion coefficient (ADC) maps of four recurrent cerebral infarctions. Panel A shows the first, panel B the second, panel C the third, and panel D the fourth cerebral infarction. (A), (C), and (D): (top) DWI shows restriction in the left pons (A, D) or right pons (C); (bottom) the ADC map shows low signal. (B): (top) DWI shows restriction in the right thalamus; (bottom left) the ADC map is isointense; (bottom right) fluid-attenuated inversion recovery shows hyperintensity, consistent with MRI findings of an infarction approximately one week after onset. Yellow arrowheads indicate the location of each infarct.

After the 56th cycle of pembrolizumab, the patient developed acute dysarthria and right hemiparesis, with a score of 2 on the US National Institutes of Health Stroke Scale [6]. Brain MRI revealed an acute infarct in the left pons (Figure 1, Panel A). Cardiac and vascular evaluations, including transthoracic echocardiography, carotid ultrasonography, and Holter monitoring, were unremarkable. He was treated in the acute phase with dual antiplatelet therapy (DAPT; clopidogrel plus aspirin) combined with intravenous argatroban and was subsequently discharged on clopidogrel monotherapy.

Within the subsequent two months, the patient developed transient numbness of the left lower lip and tongue. Brain MRI performed approximately one week after symptom onset demonstrated a lacunar infarction in the right thalamus (Figure 1, Panel B). Considering possible clopidogrel resistance, his regimen was switched to prasugrel plus aspirin. A few days later, he again developed dysarthria, and an MRI confirmed a new right pontine infarct (Figure 1, Panel C).

**Figure 2:**
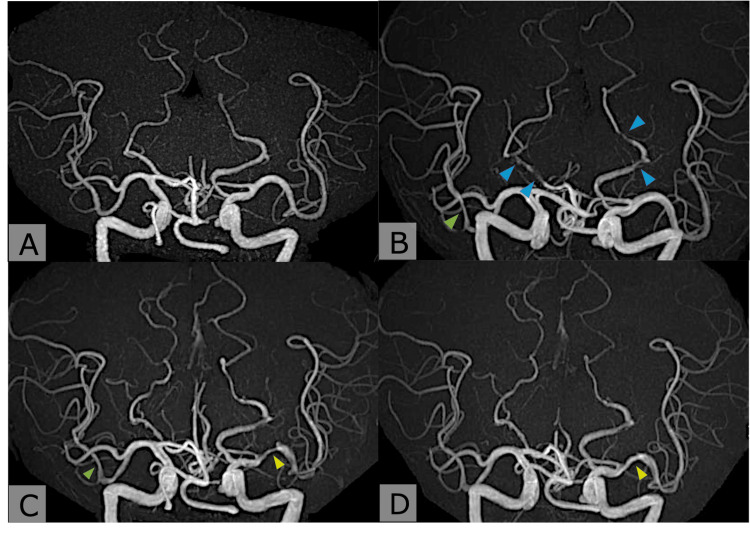
Magnetic resonance angiography (MRA) showing progression and partial improvement of intracranial arterial stenoses. (A) Brain MRA at the first admission shows no apparent arterial stenosis. (B) Multiple stenoses in the bilateral posterior cerebral arteries (PCAs) (blue arrowheads) and mild narrowing of the right middle cerebral artery (MCA) (green arrowhead). (C) Partial improvement of bilateral PCA stenoses compared to panel B; however, new stenosis is observed in the left MCA M1 segment (yellow arrowhead). (D) Improvement of the left MCA M1 segment stenosis compared to panel C (yellow arrowhead).

Several weeks later, he experienced another stroke involving the left pons (Figure 1, Panel D). MR angiography revealed new stenoses of the right middle cerebral artery (MCA) and bilateral posterior cerebral arteries (PCAs) (Figure 2, Panels A, B). Despite strict adherence to DAPT, recurrent strokes continued to occur. Extensive evaluation, including cerebrospinal fluid (CSF) studies with IL-6, antinuclear antibody, myeloperoxidase-antineutrophil cytoplasmic antibody and proteinase 3-antineutrophil cytoplasmic antibody, anti-SS-A/Ro and anti-SS-B/La antibodies, lupus anticoagulant and antiphospholipid antibody panel (anticardiolipin and β2-glycoprotein I antibodies), protein C and protein S activity, total homocysteine, and α-galactosidase A activity, was unremarkable (Table 1). Neurosyphilis relapse was excluded by both serological and CSF testing.

**Table 1:** CSF and serologic workup. CSF analysis, including IL-6, showed no abnormalities (normal cell count, protein, glucose, IgG index, and negative oligoclonal bands). Serologic testing demonstrated negative antinuclear antibody, MPO-ANCA, and PR3-ANCA, as well as negative antiphospholipid antibodies (including lupus anticoagulant), and no evident thrombophilic abnormalities on protein C, protein S, or metabolic/enzyme testing. CSF = cerebrospinal fluid; MPO-ANCA = myeloperoxidase-antineutrophil cytoplasmic antibody; PR3-ANCA = proteinase 3-antineutrophil cytoplasmic antibody

Test	Value	Units	Reference
CSF cell count	0	cells/μL	<5 cells/μL
CSF protein	49	mg/dL	10–40 mg/dL
CSF glucose	60	mg/dL	Blood glucose 98 mg/dL
IgG index	0.69	—	<0.70
oligoclonal bands	1	—	positive if ≥2 oligoclonal bands
CSF IL-6	4.4	pg/mL	<5 pg/mL
Antinuclear antibody	Negative	—	—
MPO-ANCA	<0.2	IU/mL	<3.5 IU/mL
PR3-ANCA	<0.6	IU/mL	<2.0 IU/mL
Anti-SS-A/Ro antibody	<0.4	U/mL	<0.7 U/mL
Anti-SS-B/La antibody	<0.4	U/mL	<0.7 U/mL
D-dimer	<0.5	μg/mL	<1 μg/L
Lupus anticoagulant	1.1	—	<1.2
Anticardiolipin IgG	10.5	U/mL	<20 U/mL
Anticardiolipin IgM	2.7	U/mL	<20 U/mL
β2-glycoprotein I IgG	<6.4	U/mL	<20 U/mL
β2-glycoprotein I IgM	< 1.1	U/mL	<20 U/mL
Protein C activity	162	%	70–162%
Protein S activity	108	%	63–149%
Vitamin B12	359	ng/L	233–914 ng/L
Folate	6.1	μg/L	3.6–12.9 μg/L
Total homocysteine	15.9	nmol/mL	6.3–18.9 nmol/mL
α-galactosidase A activity	56.3	nmol/mgp/h	49.8–116.4 nmol/mgp/h

**Figure 3:**
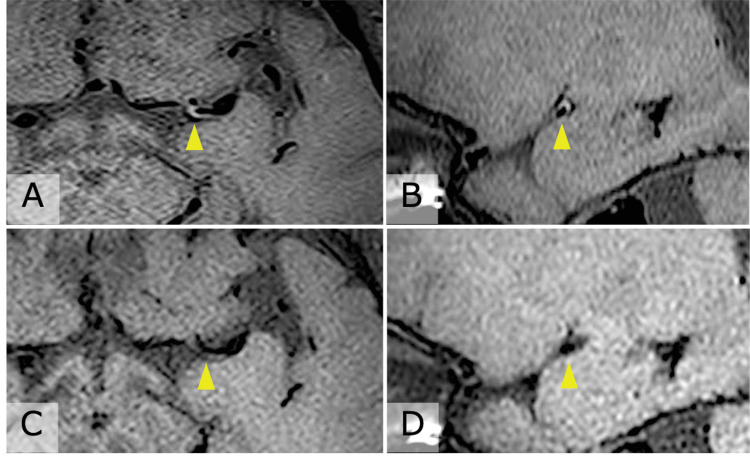
MRI black-blood sequences before and after corticosteroid therapy. (A, B) Pre-treatment images showing concentric enhancement of the left middle cerebral artery wall (yellow arrowheads). (C, D) Post-treatment images after intravenous methylprednisolone pulse therapy demonstrating resolution of vessel wall enhancement (yellow arrowheads).

**Figure 4:**
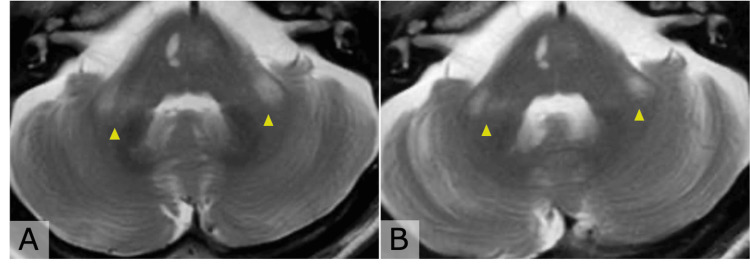
T2-weighted MRI showing reversible hyperintense lesions in the middle cerebellar peduncles before and after corticosteroid therapy. (A) Pre-treatment image demonstrating bilateral hyperintense lesions in the middle cerebellar peduncles (yellow arrowheads). (B) Post-treatment image showing a reduction in the hyperintense lesions (yellow arrowheads).

Pembrolizumab was discontinued after the 58th cycle. He remained on DAPT (prasugrel plus aspirin) for three months, followed by prasugrel monotherapy. Initially, bilateral PCA stenoses showed partial improvement; however, several months later, follow-up MRI demonstrated new stenosis of the left MCA M1 segment (Figure 2, Panel C). Vessel wall imaging (VWI) revealed concentric enhancement of the left MCA wall, and T2-weighted images showed bilateral hyperintensities in the middle cerebellar peduncles (Figure 3, Panels A, B, Figure 4, Panel A). DAPT with prasugrel and aspirin was resumed, and, because vasculitis was subsequently suspected, intravenous methylprednisolone pulse therapy (1,000 mg/day for three consecutive days) was initiated.

Three months after corticosteroid therapy, follow-up MRI showed resolution of the left MCA stenosis and vessel wall enhancement, along with reduction of the cerebellar peduncle lesions (Figure 2, Panel D, Figure 3, Panels C, D, and Figure 4, Panel B). The patient has since remained on prasugrel monotherapy without further stroke recurrence. His lung cancer has remained stable without additional pembrolizumab treatment.

## Discussion

ICIs are increasingly prescribed in oncology because of their durable efficacy against solid tumors; however, disruption of immune homeostasis can precipitate irAEs, which may be severe or life-threatening [1,2]. The incidence of nirAEs during ICI therapy is estimated at approximately 1-12%, and reported phenotypes include myasthenia gravis, myositis, encephalitis, demyelinating neuropathies, and aseptic meningitis [3,4]. Central nervous system (CNS) vasculitis related to ICIs (nirVasculitis) is rare but clinically significant. In one case of pembrolizumab-associated CNS vasculitis, malignant multifocal infarctions developed just two weeks after treatment, underscoring the potential aggressiveness of early-onset presentations [5]. A recent systematic review of nirVasculitis found that 95% of cases were associated with PD-1 inhibitors and that onset ranged widely, from soon after initiation to several months after ICI discontinuation, with late-onset events occurring as late as the 36th cycle of pembrolizumab [7]. Our patient experienced four ischemic strokes after the 56th cycle of pembrolizumab, emphasizing that nirVasculitis can also emerge after long-term treatment.

From a pathobiologic standpoint, off-target immune activation against the vascular endothelium likely promotes immune cell-mediated vasculitis, predisposing to thrombosis via coagulation factor activation and endothelial injury [8]. Prior series have reported a median time to cerebral infarction of 35 days (range = 4-222) after ICI initiation, highlighting that although early onset is common, delayed onset is also possible and clinically important [8].

Black-blood MRI (BB-MRI) or MR VWI of intracranial vessel walls is useful across disorders with differing pathophysiology, including intracranial atherosclerosis, vasculitis, dissection, and reversible cerebral vasoconstriction syndrome (RCVS) [9]. In general, vasculitic stenosis shows concentric wall thickening with contrast enhancement, whereas atherosclerosis typically exhibits focal, eccentric thickening with a mixture of an enhancing fibrous cap and a nonenhancing lipid-rich necrotic core and/or calcification. By contrast, RCVS usually demonstrates diffuse, relatively uniform wall thickening with absent or minimal enhancement. In our patient, gadolinium was contraindicated because of renal impairment, and contrast-enhanced VWI could not be performed. Noncontrast T1 BB-MRI demonstrated eccentric wall thickening at the affected segment. Although eccentric remodeling classically raises concern for atherosclerotic plaque or dissection, several features in this case supported an immune-mediated vasculitis: (i) absence of conventional atherosclerotic risk factors and no alternative embolic source; (ii) lack of imaging evidence for a lipid-rich necrotic core, calcification, an intimal flap, or a double lumen; (iii) multifocal stenoses that progressed yet were steroid-responsive on serial MRA; and (iv) radiographic reversibility, both the wall thickening and the stenoses regressed after corticosteroid therapy. In addition, bilateral T2 hyperintense lesions in the middle cerebellar peduncles (MCPs) are nonspecific but can be seen in posterior circulation vasculitis, and thus can serve as a supportive imaging marker [10,11]. Consistent with this, our patient’s bilateral MCP lesions regressed with corticosteroids, further favoring an inflammatory mechanism over neurodegenerative disease or a purely atherosclerotic, dissecting, or RCVS process. Applying the consensus disease definitions for neurologic irAEs, the overall clinical picture is most consistent with possible nirVasculitis [12].

Therapeutically, this case underscores the limitations of antithrombotic therapy alone: recurrent strokes occurred despite strict adherence to dual antiplatelet therapy, consistent with reports that antiplatelet/anticoagulant therapy is often inadequate in nirVasculitis. In contrast, discontinuation of pembrolizumab combined with high-dose corticosteroid pulse therapy produced marked radiographic and clinical improvement, highlighting the importance of both ICI withdrawal and timely immunosuppression. Given pembrolizumab’s long half-life (approximately 27 days), a prolonged steroid taper (about one to three months) is recommended to suppress ongoing inflammation [13]. In this case, the patient was taking antiviral medication for chronic hepatitis B, and, in view of the risk of HBV reactivation with prolonged high-dose steroid therapy, we did not administer oral corticosteroid maintenance therapy. Despite this, there was no relapse of vasculitis, and the clinical course was favorable. Moreover, available evidence suggests that temporary immunosuppression does not meaningfully compromise overall survival or antitumor efficacy in patients with irAEs [14], which is consistent with our patient’s stable oncologic course after ICI discontinuation.

## Conclusions

In summary, this case demonstrates that nirVasculitis can present not only early after ICI initiation but also after long-term pembrolizumab exposure, with recurrent ischemic strokes and multifocal intracranial stenoses. Management should prioritize prompt ICI cessation and immunosuppressive therapy, as antithrombotic treatment alone is insufficient. Ongoing vigilance for nirVasculitis is warranted throughout the entire course of ICI therapy, not just during the initial cycles.

## References

[1] Puzanov I, Diab A, Abdallah K (2017). Managing toxicities associated with immune checkpoint inhibitors: consensus recommendations from the Society for Immunotherapy of Cancer (SITC) Toxicity Management Working Group. J Immunother Cancer.

[2] Spain L, Diem S, Larkin J (2016). Management of toxicities of immune checkpoint inhibitors. Cancer Treat Rev.

[3] Reynolds KL, Guidon AC (2019). Diagnosis and management of immune checkpoint inhibitor-associated neurologic toxicity: illustrative case and review of the literature. Oncologist.

[4] Hottinger AF (2016). Neurologic complications of immune checkpoint inhibitors. Curr Opin Neurol.

[5] Feng J, Ross L, Ontaneda D (2021). Pembrolizumab-induced CNS vasculitis: neurologic adverse events due to checkpoint inhibitors. Neurol Clin Pract.

[6] Brott T, Adams HP Jr, Olinger CP (1989). Measurements of acute cerebral infarction: a clinical examination scale. Stroke.

[7] Erritzøe-Jervild M, Møller SN, Kruuse C, Stenør C (2025). Immune checkpoint inhibitor-related CNS vasculitis - a systematic review and report of 6 cases. J Stroke Cerebrovasc Dis.

[8] Inoue T, Kumai T, Ohara K, Takahara M (2023). Cerebral infarction as a rare adverse event of immune checkpoint inhibitors in patients with head and neck squamous cell carcinoma: a case series. Cureus.

[9] Gomyo M, Tsuchiya K, Yokoyama K (2023). Vessel wall imaging of intracranial arteries: fundamentals and clinical applications. Magn Reson Med Sci.

[10] Morales H, Tomsick T (2015). Middle cerebellar peduncles: magnetic resonance imaging and pathophysiologic correlate. World J Radiol.

[11] Okamoto K, Tokiguchi S, Furusawa T, Ishikawa K, Quardery AF, Shinbo S, Sasai K (2003). MR features of diseases involving bilateral middle cerebellar peduncles. AJNR Am J Neuroradiol.

[12] Guidon AC, Burton LB, Chwalisz BK (2021). Consensus disease definitions for neurologic immune-related adverse events of immune checkpoint inhibitors. J Immunother Cancer.

[13] (2025). KEYTRUDA® (pembrolizumab). https://www.pmda.go.jp/drugs/2016/P20161025002/170050000_22800AMX00696000_G100_1.pdf.

[14] Horvat TZ, Adel NG, Dang TO (2015). Immune-related adverse events, need for systemic immunosuppression, and effects on survival and time to treatment failure in patients with melanoma treated with ipilimumab at Memorial Sloan Kettering Cancer Center. J Clin Oncol.

